# Semi-Arid Environmental Conditions and Agronomic Traits Impact on the Grain Quality of Diverse Maize Genotypes

**DOI:** 10.3390/plants13172482

**Published:** 2024-09-05

**Authors:** Nicolás Francisco Bongianino, María Eugenia Steffolani, Claudio David Morales, Carlos Alberto Biasutti, Alberto Edel León

**Affiliations:** 1 Córdoba Food Science and Technology Institute (ICYTAC), National Scientific and Technical, Research Council (CONICET), National University of Córdoba (UNC), Córdoba 5000, Argentina; eusteffolani@agro.unc.edu.ar (M.E.S.); david.morales@unc.edu.ar (C.D.M.); aeleon@agro.unc.edu.ar (A.E.L.); 2 Plant Breeding, College of Agricultural Sciences, National University of Córdoba (UNC), Córdoba 5000, Argentina; biasutti@agro.unc.edu.ar; 3 Biological Chemistry, College of Agricultural Sciences, National University of Córdoba (UNC), Córdoba 5000, Argentina

**Keywords:** food security, climate change, genetic improvement, variance components, maize quality

## Abstract

We assessed the impact of environmental conditions and agronomic traits on maize grain quality parameters. The study was conducted using genotypes with distinct genetic constitutions developed specifically for late sowing in semi-arid environments. We evaluated the agronomic, physical, and chemical characteristics of eight maize open-pollinated varieties, six inbred lines, and three commercial hybrids. The yield of the open-pollinated varieties showed a positive correlation with protein content (r = 0.33), while it exhibited a negative correlation with the carbohydrate percentage (r = −0.36 and −0.42) in conjunction with the inbred lines. The flotation index of the hybrids was influenced primarily by the environmental effect (50.15%), whereas in the inbred lines it was nearly evenly divided between the genotype effect (45.51%) and the environmental effect (43.15%). In the open-pollinated varieties, the genotype effect accounted for 35.09% and the environmental effect for 42.35%. The characteristics of plant structure were associated with grain quality attributes relevant for milling, including hardness and test weight. Inbred lines exhibited significant genotype contributions to grain hardness, protein, and carbohydrate content, distinguishing them from the other two germplasm types. These associations are crucial for specific genotypes and for advancing research and development of cultivars for the food industry.

## 1. Introduction

Maize is cultivated in over 160 countries across different agro-climatic zones [1]. This versatile grain can be used to produce food, animal feed, and various industrial applications [2].

Climate change is a widely studied phenomenon caused by increased industrial activity and the emission of greenhouse gases into the atmosphere [3]. This is leading to changes in precipitation and the hydrological cycle [4]. The climate changes could lead to longer droughts and more intense precipitation, affecting groundwater levels due to variations in irrigation demand and water profile fluctuations [5]. The world’s food security is at risk due to these changes, which are affecting food production, quality, prices, and supply chains [6].

Reduction in nutrient availability is an aggravating factor in the phenological cycle of crops, which is shortened by all unfavorable conditions (irregular rainfall, higher temperatures, and variations in daylight hours) [6]. As a result, grains from cereals like rice, wheat, and maize have lower yields and a poorer nutritional composition, which has an immediate impact on the quality of food made from them [7,8,9].

Despite challenging growing conditions, climate adaptation is essential for improving crop performance. Enhancing production stability also requires careful consideration of crop calendar management, strategic variety selection, better irrigation, and waste management [10,11]. Climate change impacts the amount and distribution of rainfall in Argentina’s semi-arid central region, influencing maize productivity. Breeding programs must therefore take into account the adaptation of genotypes to these unique conditions [12]. Similarly, evaluating the effects of the environment on genotypes is crucial in these programs, as this can provide information on the assessment of the stability and adaptability of the final genotypes or base populations. In this context, it is important to keep in mind that this may imply that a superior crop variety in one environment may not necessarily perform best in another [13]. Assessing various genotypes for quality traits linked to yield can aid in developing efficient breeding methods for the future [14]. Furthermore, adding value to raw materials will boost the rural economy [15].

There is little information on how climatic conditions and agronomic characteristics affect grain physical and chemical parameters simultaneously evaluated in inbred lines, hybrids, and open-pollinated varieties. Thus, this study aimed to evaluate the effect of environmental conditions and agronomic traits on maize grain quality parameters in inbred lines, hybrids, and open-pollinated varieties developed for late sowing in semi-arid environments.

## 2. Results and Discussion

### 2.1. Climate Characterisation

The cumulative rainfall during the 2018 and 2020 crop cycles was below the historical average (489 mm) for this region, with 392 mm and 462 mm. Furthermore, when considered globally, there was a significant decrease in rainfall during the critical periods (cp) (late January–early April) for the genotypes, with 64.25 mm and 71.36 mm. Huang et al. [16] mentioned that understanding crop-specific water requirements enabled effective sowing time management, and alterations in rainfall patterns significantly affected crop yield.

Based on the average temperature fluctuations during the cropping seasons, the highest mean and maximum temperatures occurred in the critical periods of 2018 and 2020. Kothiyal and Kaur [17] highlighted that elevated temperatures significantly affect crop physiological processes, including respiration, photosynthesis, and photoassimilate partitioning. Thus, yield reductions in maize may occur with a temperature increase of up to 1 °C–2 °C.

### 2.2. Agronomical Traits

Among the hybrid group (Table 1), the P2089 material stood out due to its taller average height of 2.22 m, with the main ear positioned 0.86 m above the soil surface. This cultivar also showed statistical similarities with AX882 and P1815 in ear diameter (4.49 cm, 4.50 cm, and 4.19 cm, respectively). The P2089 genotype outperformed P1815 in grain yield, showing values of 8083.48 kg/ha and 5806.34 kg/ha, respectively. These yields are similar to the average yield from this productive region in recent years (7100 kg/ha) [18]. The inbred lines presented plant heights between 1.71 m (C4B) and 2.06 m (CIM06), a spike insertion height between 0.76 m (C4B) and 1.00 m (CIM06), and a stem diameter between 1.87 cm (B4) and 2.86 cm (BCOT). In addition, the BCOT genotype (5208.57 kg/ha) contrasted significantly with C4B (2422.41 kg/ha) in grain yields. The OPV varieties (C8008 and BlancoM) had a higher average plant size and ear diameter in all growing seasons. Conversely, C980 and C990 displayed some of the highest average yields, with 4451.85 kg/ha and 4469.00 kg/ha, respectively, in comparison with genotypes such as C8008. The latter reached an average yield for all growing seasons of 2624.75 kg/ha. These results are consistent with those published by Sibanda et al. [19], who indicated that values ranged from 2970 kg/ha to 5040 kg/ha for OPVs with a medium yield potential.

### 2.3. Grain Physical–Chemical Characteristics

Regarding the weight of 1000 grains (Table 2) analyzed in the hybrids, the genotype AX882 (310.16 g) showed a significant superiority over P1815 (243.39 g). Furthermore, the genotype P1815 was superior to the other hybrids in terms of ash content (1.74%). The L cultivars B4 and BL04 had the lowest grain hardness, as indicated by their highest flotation index values (62.13% and 64.5%, respectively). Furthermore, the genotype B4 exhibited the highest W1000. However, these same lines diverged significantly in grain chemical composition. Both B4 and BL04 obtained the lowest protein content values (9.42% and 9.66%, respectively), yet they showcased the highest expression in terms of carbohydrate percentage (84.98% and 85.55%). Sharma and Carena [15] pointed out that the chemical composition, including protein, oil and total starch content, is predominantly influenced by additive gene action. Furthermore, they observed a significant correlation between parental and offspring genotypes for these traits, which intensifies under extreme environmental conditions. This suggests that data from inbred lines could predict hybrid performance across varying environments.

The open-pollinated varieties (OPVs) BlancoM and C8008 demonstrated a superior performance in several key metrics. BlancoM exhibited the highest flotation index at 79.5%, followed by C8008 with 60.13%. In terms of grain weight, BlancoM also had the highest value for a weight of 1000 grains at 342.32 g. However, the C8008 variety showed the lowest test weight values, with C8008 at 80.84 kg/HL as detailed in Table 2. Regarding nutritional content, the OPVs displayed protein contents ranging from 9.05% to 10.67%, lipids contents between 4.27% and 5.74%, and carbohydrates between 81.80% and 84.80%. Ash content showed minimal variation across all harvest years, with values from 1.64% to 1.79%. Kljak et al. [20] mentioned that a higher number of floating grains indicates lower hardness, vitreousness percentage, and breakage susceptibility. The hardness of the grain is explained by the arrangement of starch granules within the protein matrix of the grain endosperm. The mealy endosperm is characterized by loosely organized granules in a thinner protein matrix with multiple air-filled spaces, while glassy endosperm contains tightly organized granules. Environmental factors before harvest and post-harvest conditions such as handling, transport, drying, storage, and processing can influence grain hardness; however, it is primarily determined by the genetic expression unique to each variety [21]. Nguma et al. [22] noted that certain types of zein are associated with grain hardness; specifically, jagged grains contain higher levels of γ-zein synthesized in the mealy fraction of the endosperm’s inner core [23].

Grain physical aspects, such as the flotation index and weight of 1000 grains (W1000), were negatively associated with mean yield across all growing seasons, as indicated in Table 2. The flotation index was correlated with yield, showing r coefficients of −0.39 for L and −0.28 for OPV. Additionally, the W1000 values ranged between −0.62 and −0.83 for the three material types examined. Nemati et al. [24] reported that the 1000 grain weight (TGW) had no significant effect on yield in early planting when a longer growing season is evident. This contrasts with Jamshidian et al. [25], who suggested that indirect selection for traits like TGW can improve ear yield, especially in early generations of dent maize hybrids. Cerrudo et al. [26] further highlighted that maize grain hardness was independent of yield but rather dependent on the availability of photoassimilates per unit grain. They proposed that a combination of increased photosynthetic activity and reduced grain number due to growth restriction could ultimately reduce yield while increasing grain hardness.

The protein content in OPV showed a positive correlation with yield (r = 0.33). This suggests that increasing nitrogen uptake or maintaining high nitrogen partitioning within the grain can lead to higher protein [27]. However, Šeremešić et al. [28] found that modern hybrids exhibited a different response of grain protein content to plant density and environment compared to older varieties. Inbred lines and OPV exhibited negative correlations between carbohydrate content and yield, with r correlation coefficients of −0.42 and −0.36, respectively (Table 2). Amegbor et al. [29] reported significant positive associations between grain yield and protein content, while starch content was negatively associated, which is in line with current trends. Additionally, lipid content showed a strong positive correlation for L, with an r-value of 0.76. Ramana Reddy et al. [30] also demonstrated a positive relationship between grain yield and lipid content for both inbred lines and hybrid crosses, with values of 0.35 and 0.52, respectively. However, other researchers have observed that the development of increasingly productive hybrid corn varieties has led to a decrease in nutritional value, particularly in protein and lipid content [31].

The thermal time required to reach the phenological flowering stage was found to be significantly and positively correlated with grain physical parameters, such as the weight of 1000 grains (r = 0.37, *p* = 0.0001) and the flotation index (r = 0.29, *p* = 0.0016). Conversely, a negative correlation was observed with protein content (r = −0.30, *p* = 0.0011). Butts-Wilmsmeyer et al. [32] observed that the accumulation of storage materials such as proteins and starch during grain development is influenced by temperature and soil moisture, which in turn affects yield and grain composition. Similarly, Chen et al. [33] demonstrated that drought stress leads to reduced starch accumulation in the endosperm, significantly contributing to a decrease in grain weight [34]. Jahangirlou et al. [35] highlighted that resource availability, particularly water and nitrogen, significantly impacts germ growth and the enzymatic activity involved in lipid biosynthesis. They found that water availability during the critical period (cp) was positively correlated with both the grain test weight (r = 0.21, *p* = 0.0290) and lipid content (r = 0.29, *p* = 0.0018). Additionally, plants undergoing flowering have mechanisms to withstand stress by accelerating this phase to expedite flower and seed production [36]. Moreover, certain plants have evolved drought-tolerance mechanisms that enhance water-use efficiency [37].

Maximum temperatures were found to be positively correlated with W1000 (r = 0.62, *p* < 0.0001 and FI (r = 0.36, *p* = 0.0001), while showing a negative correlation with TW (r = −0.39, *p* < 0.0001). These temperatures also correlated with a higher percentage of carbohydrates (r = 0.49, *p* = 0.0001) and a lower lipid content (r = −0.54, *p* < 0.0001). Significant correlations were also observed between the grain’s physical characteristics and its chemical composition. Notably, protein content was negatively correlated with both the flotation index (r = −0.72, *p* < 0.0001) and W1000 (r = −0.27, *p* = 0.0033). Lipid content showed an inverse relationship with W1000 (r = −0.36, *p* = 0.0001) and a direct relationship with TW (r = 0.51, *p* < 0.0001). Carbohydrate content exhibited significant correlations with all three physical parameters: a negative correlation with hectoliter weight (r = −0.39, *p* < 0.0001), and positive correlations with P1000 (r = 0.47, *p* < 0.0001) and IF (r = 0.69, *p* < 0.0001). Narváez-González et al. [38] indicated that the compactness of endosperm cell bodies determines the physical properties of the grain, including weight, size, hard endosperm percentage, protein content, and pericarp thickness.

**Figure 1:**
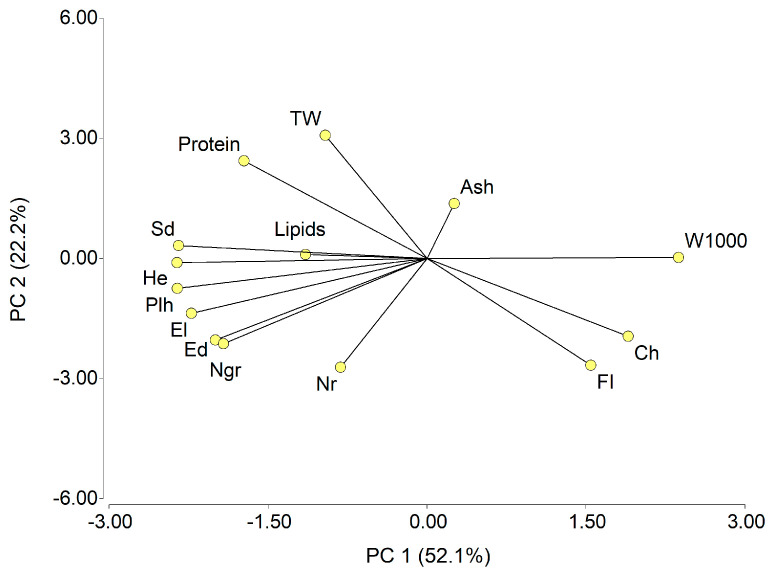
Principal component analysis for grain physical–chemical parameters and agronomic characteristics. W1000, weight of a thousand grains; TW, test weight; FI, flotation index; Ch, carbohydrates; Plh, plant height; He, height of insertion of the main ear; Sd, stem diameter; El, ear length; Ed, ear diameter; Nr, number of rows; Ngr, number of grains per row.

The associations between the physical–chemical parameters of the grain and the agronomic characteristics of the genotypes suggest that variability can be largely explained by two principal components: PC1 (52.1%) and PC2 (22.2%), accounting for 85.7% of the total variation (Figure 1). The weight of W1000 and the flotation index are inversely related to all of the structural parameters of the plant. Among the chemical parameters, protein content showed the strongest relationship with genotype-specific traits, particularly those related to vegetative structure and ear characteristics. Conversely, carbohydrate percentage showed a negative correlation with these agronomic traits, while lipid content displayed a similar pattern to that of proteins. Grain hardness, an intrinsic property of genotypes, is negatively associated with starch content but positively correlated with amylose levels [39]. However, environmental factors can influence the starch biosynthetic pathway, affecting enzymes involved in substrate production, the elongation of α-1,4-glucan chains, branching, and maintenance of the granules’ crystalline structure [39,40].

### 2.4. Variance Components for Grain Physical–Chemical Characteristics

Firstly, it was observed that the flotation index varied among the materials evaluated (Table 3). Environmental effects accounted for 50.15% of the variability in flotation index expression for H, while the variability was nearly evenly split between genotype effects (45.51%) and environmental effects (43.15%) for inbred lines. In OPVs, genotype effects represented 35.09% of the variability and environmental effects accounted for 42.35%. This important incidence of the genotype effect on the indirect expression of grain hardness (flotation index) coincides—although with a lower value—with that reported by Caballero-Rothar et al. [41], who indicated a contribution of 88% of the total variability. For W1000, the explanation of the variability between materials was very similar, since the environmental effect obtained the highest participation, with values between 84.33% and 92.80%. Similarly, the effect of genotype and the GxE interaction was very small or null for TW; thus, the environmental effect obtained a high percentage in the three types of germplasm.

The expression of protein character in the lines was influenced almost equally by genotype effects and genotype–environment interactions, with variance percentages of 45.9% and 41.77%, respectively (Table 3). In contrast, environmental factors played a more dominant role in the variance components for open-pollinated varieties (OPVs), despite having a significant 58.54% influence on hybrids. Previous studies [42,43] have highlighted the substantial impact of environmental factors on protein expression. Regarding lipid content, environmental factors and their interaction with genotype were the most influential, with the genotype explaining only a minor percentage of the variability in OPVs. The significance of the environmental–genotype interaction on lipid content is supported by findings from Fang et al. [44]. While genetics plays a major role in determining lipid concentration in maize grain, environmental conditions and agronomic practices such as fertilization and planting density can also induce variations [45]. Ndlovu et al. [46] noted that nitrogen levels in soil could affect protein content and the saturated/unsaturated fatty acid ratio in grain lipids. Ngaboyisonga and Njoroge [47] found that certain inbred lines produced more stable offspring with higher grain hardness, protein quantity, and quality under low-nitrogen drought conditions. The influence of the male gamete (pollen) on lipid content suggests a paternal effect without impacting seed weight, unlike protein content which is solely affected by maternal factors [48,49,50]. Ash content was represented by genotype effects (29.95%) and environmental effects (35.45%) in hybrids, while in inbred lines and OPVs these components poorly explained this trait (Table 3). The effect of the environment on the material evidenced less stability in expressing its attributes when a character demonstrated a significant GxE interaction, as noted by Etiro et al. [51].

Özdemir and Sade [52] stated that trait variance components depend on the genetic makeup of the population, with different genetic effects influencing trait inheritance. The additive component contributes to phenotype expression, while “missing heritability” may be due to epistasis, phenotypic plasticity, or rare genetic variants [53]. Al-Naggar et al. [54] demonstrated the importance of additive components over non-additive effects since the general combining ability (GCA) exceeded the specific combining ability (SCA) for grain protein, oil, and starch content. To improve this type of character in materials with wide genetic variability, an effective approach is often the recurrent selection of half-sib families [55]. Thus, a significant additive gene action effect in a specific population suggests that early-generation selection may result in the development of transgressive homozygous lines [56].

## 3. Materials and Methods

### 3.1. Genetic Material and Experimental Design

We used three commercial hybrids (“AX882” and “P1815” yellow-soft grain; “P2089” yellow-very soft grain), six inbred lines obtained by self-fertilization in inbreeding cycles at the Campo Escuela of the Facultad de Ciencias Agropecuarias, UNC (“B4” and “BL04” yellow-soft grain; “BCOT”, “BulkASC”, “C4B”, and “CIM06” yellow-hard grain), and eight open-pollinated maize varieties developed (“BlancoM” white-soft grain; “C6006” orange-hard grain; “C8008” white-intermediate grain; “C900”, “C980”, “C990”, “CandelariaINTA”, and “LealesINTA” orange-intermediate grain) by selection for adaptation to the semi-arid central zone of Argentina. The genotypes were arranged in a randomized complete block design (RCBD) with two replications in experimental plots in the Campo Escuela (FCA, UNC) located in the semi-arid central region of Argentina (31°29′ S; 64°00′ W). The sowing dates were established in mid-December during the 2017–2018, 2018–2019, 2019–2020, and 2020–2021 seasons (Table 4). Conventional tillage (CT) was used for the first two seasons and no-till (NT) was used for the rest of the seasons. The experiments were conducted under rainfed conditions. The soil is an Entic Haplustoll and the superficial horizon presents a silt-loam texture, slightly acid to neutral, and well-supplied organic matter and is well-drained. Also, it does not present impediments that limit the crop growth.

### 3.2. Agronomical Traits

Agronomical traits included the following: plant height (Plh), recorded from the base of the stem to the apical end of the extended panicle (m); height of insertion of the main ear (He), recorded from the base of the stem to the node of the upper ear (most apical position) (m); stem diameter (Sd), recorded at the mean height of the first internode from the distal end of the stem (cm); ear length (El), recorded from distal to apical through the middle part of the spike (cm); ear diameter (Ed), measured in the middle part of the ear (cm); number of rows (Nr), recorded in the middle part of the ear; number of grains per row (Ngr), recorded from distal to apical in randomly selected rows; yield (Y), estimated using the production of each experimental plot and extrapolated to the area equivalent to one hectare (10,000 m^2^). The yield results were adjusted to 14% humidity and expressed in quintals per hectare (q/ha). The traits were recorded on randomly selected plants until they reached 50% of the total plants in each plot. Thermal time to flowering (TT) was estimated from a base temperature of 10 °C. Climate data were collected during the crop critical period (anthesis ± 15 days) and included the minimum, average, and maximum temperatures as well as accumulated precipitation. These parameters were obtained from an agrometeorological station (Omixom SRL, Córdoba, Argentina) installed in this establishment.

### 3.3. Grain Physical Properties

The weight of 1000 grains (W1000) was calculated using an analytical balance [57]. The test weight (TW) was estimated using a Schopper balance of 250 mL total volume with a piston for air displacement [58]. The hardness of the grain was determined indirectly by the flotation index (FI) with a sodium nitrate solution (density (q) 1.250 ± 0.001) [59].

### 3.4. Flour Chemical Traits

Whole grain samples were ground using a Cyclotec CT193 cyclone mill (Foss, Suzhou, China) with a mesh aperture of 1 mm. The proximate composition was determined through proteins, lipids, and ash according to Methods 46-10.01, 30-25.01, and 08-01.01, respectively (AACC-approved methods of analysis, 2010). The carbohydrate content was estimated by the difference between the sum of the other components (proteins, lipids, and ashes). All determinations were expressed in g per 100 g of sample on a dry basis and were performed in duplicate.

### 3.5. Statistical Analysis

The InfoStat/Professional 2022 software (Facultad de Ciencias Agropecuarias, Universidad Nacional de Córdoba) and R 3.6.3 software (R Foundation for Statistical Computing, Vienna, Austria) were used. The data were examined using analysis of variance (ANOVA), with a significance level of 0.05. In addition, an LSD Fisher test for environmental parameters and a Tukey’s HSD mean comparison test for agronomic, grain physical, and flour chemical traits, considering all harvest campaigns, were carried out [60,61]. Separate analyses were carried out according to the type of genetic material (OPV, L, and H). To appreciate the effects of the different sources of variation on the total variability, the variance components were estimated using Mixed Linear Models (MLMs) [62,63] using the restricted maximum likelihood estimator (REML) [64,65]. Thus, the MLM model_X_REML < −lmer(X~1 + (1|Genotype) + (1|Environment) + (1|Genotype: Environment) with the lme4 statistical package version 1.1–35.5 was used, where X indicates the trait under study. The components of genotype, environment, and their interaction were considered random effects. Regarding the environmental factor, the crossing between the year and the tillage method used (conventional tillage and direct sowing), was considered. In this way, it was possible to obtain the magnitude of each variance (genotypic, environmental, and genotype–environment interaction) calculated as a percentage of the total variation. The relationships between the analyzed variables were determined using the Pearson correlation test, with a significance level of *p* ≤ 0.05 and 0.01.

## 4. Conclusions

The intrinsic characteristics of the plant are essential to determine milling quality attributes such as grain hardness (measured indirectly by flotation index) and test weight. In addition, specific environmental factors from the semi-arid areas, such as rainfall and temperature, have a significant effect on grain composition in open-pollinated varieties, inbred lines, and hybrids. In particular, protein and lipid contents tend to decrease with higher temperatures during the critical growth period, while carbohydrate contents increase.

Inbred lines show significant genotype contributions to flotation index, protein, and carbohydrate content, which distinguishes them from the other two germplasm types. Identifying stability and sensitivity of specific traits—such as grain physical aspects and chemical composition—is crucial when selecting materials for potential improvement in variety development, targeting areas with stress conditions. This allows us to identify production environments where desired traits are optimally expressed.

The yield of the evaluated inbred lines was associated with a higher test weight and a lower grain weight and flotation index. Yield was also associated with higher lipid and lower carbohydrate contents. Considering the importance of ensuring food security in the current context of climate change, it is necessary to develop new crop varieties capable of producing high-quality raw materials in sufficient quantities for the industry. In this sense, the results of this study have identified specific genotypes, such as the inbred lines BL04 and B4, which are well-adapted to semi-arid conditions and have the potential to produce hybrids with high carbohydrate contents. In addition, inbred lines such as C4B, BulkASC, and BCOT can be used to produce hybrids with good protein contents.

## Figures and Tables

**Table 1:** Growing season average values for agronomic characteristics of hybrids, inbred lines, and open-pollinated varieties.

Genotype	Ger.	Plh (m)	He (m)	Sd (cm)	Ed (cm)	Nr	Ngr	El (cm)	TT (°C d)	Yield (kg/ha)
AX882	H	1.94 ± 0.36 a	0.71 ± 0.19 a	2.03 ± 0.39 a	4.50 ± 0.55 a	15.00 ± 1.68 a	31.20 ± 6.22 a	14.89 ± 2.31 a	946.20 ± 81.23 a	7071.10 ± 4741.44 ab
P1815		2.02 ± 0.43 a	0.79 ± 0.35 b	2.13 ± 0.46 a	4.19 ± 0.58 a	14.88 ± 1.96 a	29.85 ± 8.74 a	14.01 ± 2.98 a	1044.05 ± 83.75 b	5806.34 ± 3896.20 a
P2089		2.22 ± 0.50 b	0.86 ± 0.29 b	2.14 ± 0.49 a	4.49 ± 0.61 a	15.13 ± 2.13 a	32.85 ± 10.76 a	14.85 ± 3.90 a	1047.28 ± 73.53 b	8083.48 ± 5307.75 b
B4	L	1.80 ± 0.23 ab	0.87 ± 0.22 ab	2.44 ± 0.45 a	4.03 ± 0.56 a	14.25 ± 1.61 a	25.45 ± 1.24 ab	14.09 ± 2.11 a	979.28 ± 73.26 d	3297.58 ± 1137.80 ab
BCOT		2.00 ± 0.59 ab	1.00 ± 0.25 b	2.85 ± 0.18 c	3.88 ± 0.72 a	13.55 ± 1.65 a	34.00 ± 0.00 c	14.63 ± 3.67 a	854.63 ± 15.79 ab	5208.57 ± 3666.23 b
BL04		1.87 ± 0.51 ab	0.96 ± 0.12 ab	2.47 ± 0.15 b	3.72 ± 0.61 a	13.8 ± 1.48 a	25.50 ± 1.84 ab	13.80 ± 3.26 a	890.78 ± 57.53 c	3183.14 ± 1503.38 ab
BulkASC		1.97 ± 0.47 ab	0.95 ± 0.13 ab	2.46 ± 0.04 b	3.95 ± 0.85 a	14.25 ± 2.22 a	25.70 ± 1.27 ab	12.62 ± 1.80 a	879.90 ± 32.79 bc	3035.02 ± 1364.56 ab
C4B		1.71 ± 0.25 a	0.76 ± 0.17 a	2.52 ± 0.08 b	3.42 ± 0.34 a	12.40 ± 0.57 a	21.10 ± 2.40 a	11.98 ± 2.22 a	957.95 ± 26.33 d	2422.41 ± 738.69 a
CIM06		2.06 ± 0.46 b	0.89 ± 0.18 ab	2.59 ± 0.08 bc	3.63 ± 0.78 a	13.65 ± 1.68 a	27.90 ± 2.69 bc	15.41 ± 3.75 a	846.53 ± 25.14 a	4064.77 ± 2843.87 ab
BlancoM	OPV	2.27 ± 0.59 ab	1.17 ± 0.36 c	2.43 ± 0.50 bc	4.34 ± 0.52 b	12.50 ± 0.53 a	27.50 ± 7.34 b	15.58 ± 3.49 a	1268.73 ± 125.56 b	3188.54 ± 2110.93 ab
C6006		2.09 ± 0.49 ab	0.99 ± 0.27 ab	2.26 ± 0.49 abc	4.01 ± 0.33 a	12.85 ± 1.50 a	25.40 ± 4.13 ab	14.35 ± 2.08 a	1111.28 ± 68.29 a	3940.18 ± 2004.07 ab
C8008		2.25 ± 0.56 b	1.13 ± 0.36 bc	2.50 ± 0.48 c	4.35 ± 0.50 b	13.48 ± 1.51 a	25.90 ± 8.07 ab	15.08 ± 2.67 a	1300.15 ± 104.91 b	2624.75 ± 1736.43 a
C900		2.10 ± 0.44 ab	1.01 ± 0.27 abc	2.19 ± 0.48 ab	3.98 ± 0.39 a	12.83 ± 1.53 a	25.95 ± 7.80 ab	14.24 ± 2.88 a	1111.50 ± 70.11 a	3574.68 ± 1897.84 ab
C980		2.10 ± 0.41 ab	0.96 ± 0.30 ab	2.37 ± 0.47 abc	4.11 ± 0.34 ab	13.10 ± 0.95 a	26.50 ± 2.83 ab	15.10 ± 2.27 a	1121.73 ± 77.85 a	4451.85 ± 2168.74 b
C990		2.17 ± 0.44 ab	1.03 ± 0.28 abc	2.23 ± 0.39 abc	4.05 ± 0.44 ab	13.25 ± 0.92 a	25.05 ± 8.08 ab	14.19 ± 2.82 a	1137.95 ± 70.54 a	4469.00 ± 2014.61 b
CandelariaINTA		2.13 ± 0.48 ab	0.95 ± 0.31 a	2.28 ± 0.45 abc	4.19 ± 0.36 ab	13.65 ± 1.09 a	28.55 ± 11.85 ab	14.89 ± 3.36 a	1125.26 ± 62.03 a	4244.14 ± 2731.87 ab
LealesINTA		2.01 ± 0.37 a	0.96 ± 0.26 ab	2.08 ± 0.42 a	4.25 ± 0.38 ab	14.14 ± 1.02 a	23.60 ± 6.48 a	13.94 ± 2.81 a	1131.66 ± 60.92 a	3077.72 ± 1846.28 ab

Average values (± standard deviation) where different letters on each column and genotype group indicate statistical differences (Tukey’s HSD test, *p* ≤ 0.05). Ger., type of germplasm; H, hybrid; L, inbred line; OPV, open-pollinated varieties; Plh, plant height; He, height of insertion of the main ear; Sd, stem diameter; Ed, ear diameter; Nr, number of rows; Ngr, number of grains per row; El, ear length; TT, thermal time to flowering.

**Table 2:** Growing season average values for physical–chemical grain characteristics and their correlations with yield in hybrids, inbred lines, and open-pollinated varieties.

Genotype	Ger.	FI (%)	Hardness	W1000 (g)	TW (kg/HL)	Protein (%)	Lipids (%)	Ch (%)	Ash (%)
AX882	H	83 ± 14.89 a	Soft	310.16 ± 48.86 b	82.83 ± 4.81 a	8.05 ± 1.3 a	4.68 ± 0.42 a	85.62 ± 1.43 a	1.65 ± 0.09 a
P1815		86.63 ± 11.53 a	Soft	243.39 ± 60.23 a	83.63 ± 7.68 a	7.71 ± 0.57 a	5.03 ± 1.72 a	85.51 ± 1.7 a	1.74 ± 0.11 b
P2089		90.38 ± 6.86 a	Very Soft	289.66 ± 63.13 ab	82.13 ± 6.54 a	7.38 ± 0.84 a	4.71 ± 0.24 a	86.29 ± 0.98 a	1.62 ± 0.05 a
Yield		n/s		−0.83 **	n/s	n/s	n/s	n/s	−0.68 **
B4	L	62.13 ± 15.98 b	Soft	325.09 ± 34.68 c	86.13 ± 5.29 a	9.42 ± 1.12 a	3.92 ± 0.7 a	84.98 ± 1.28 c	1.68 ± 0.15 ab
BCOT		22.25 ± 9.54 a	Hard	270.84 ± 51.4 b	93.4 ± 0.28 a	12.05 ± 1.59 b	4.54 ± 2.94 a	81.56 ± 1.41 a	1.84 ± 0.09 b
BL04		64.5 ± 23.74 b	Soft	271.44 ± 38.76 b	87.8 ± 0.28 a	9.66 ± 0.33 a	3.28 ± 0.51 a	85.55 ± 0.8 c	1.51 ± 0.04 a
BulkASC		25.75 ± 20.17 a	Hard	262.73 ± 62.26 b	85.25 ± 9.77 a	12.19 ± 1.13 b	3.42 ± 1.8 a	82.63 ± 0.72 ab	1.76 ± 0.05 ab
C4B		21.88 ± 18.88 a	Hard	189.59 ± 55.06 a	95.5 ± 0.14 a	12.53 ± 1.09 b	3.31 ± 1.38 a	82.50 ± 0.56 ab	1.66 ± 0.27 ab
CIM06		19.75 ± 10.9 a	Hard	262.44 ± 82.94 b	85.45 ± 8.17 a	11.15 ± 0.21 ab	3.77 ± 0.09 a	83.45 ± 0.12 b	1.63 ± 0.01 ab
Yield		−0.39 *		−0.62 **	0.63 **	n/s	0.76 **	−0.42 *	n/s
BlancoM	OPV	79.5 ± 16.78 c	Soft	342.32 ± 48.42 c	82.08 ± 6.92 ab	9.18 ± 0.75 a	4.27 ± 1.1 a	84.80 ± 1.6 c	1.76 ± 0.25 a
C6006		30.63 ± 23.35 a	Hard	279.03 ± 37.51 ab	87.35 ± 4.67 c	9.45 ± 1.21 ab	4.44 ± 0.93 a	84.42 ± 1.27 bc	1.69 ± 0.09 a
C8008		60.13 ± 11.31 b	Intermediate	303.41 ± 66.67 b	80.84 ± 7.63 a	9.78 ± 0.81 ab	4.6 ± 1.1 ab	83.94 ± 1.66 bc	1.68 ± 0.09 a
C900		39.5 ± 16.86 a	Intermediate	277.96 ± 46.79 ab	86.8 ± 6.1 bc	10.19 ± 0.88 ab	5.01 ± 0.66 ab	83.04 ± 1.24 ab	1.75 ± 0.08 a
C980		38 ± 14.07 a	Intermediate	254.88 ± 41.76 a	87.35 ± 5.64 c	9.77 ± 0.74 ab	4.44 ± 0.89 a	84.07 ± 0.73 bc	1.71 ± 0.12 a
C990		45.63 ± 21.61 ab	Intermediate	270.66 ± 43.05 ab	86.65 ± 6.9 bc	9.86 ± 0.64 ab	5.11 ± 1.56 ab	83.31 ± 1.88 abc	1.72 ± 0.12 a
CandelariaINTA		40.38 ± 16.05 a	Intermediate	263.59 ± 34.05 a	84.38 ± 5.31 abc	9.05 ± 0.98 a	4.52 ± 0.63 a	84.79 ± 0.82 c	1.64 ± 0.05 a
LealesINTA		39.75 ± 24.71 a	Intermediate	257.59 ± 39.43 a	84.14 ± 5.7 abc	10.67 ± 1.76 b	5.74 ± 1.52 b	81.80 ± 2.55 a	1.79 ± 0.17 a
Yield		−0.28 *		−0.8 **	n/s	0.33 **	n/s	−0.36 **	n/s

Average values (±standard deviation) where different letters on each column and genotype group indicate statistical differences (Tukey’s HSD test, *p* ≤ 0.05). * and ** indicate significance at 95 and 99%, respectively. n/s means not significant. Ger., type of germplasm; H, hybrid; L, inbred line; OPV, open-pollinated varieties; FI, flotation index; W1000, weight of a thousand grains; TW, test weight; Ch, carbohydrates.

**Table 3:** Variance components for physical–chemical grain parameters evaluated in hybrids, inbred lines, and open-pollinated varieties for all growing seasons.

Genotype	VC (%)	FI (%)	W1000 (g)	TW (kg/HL)	Protein (%)	Lipids (%)	Ch (%)	Ash (%)
Hybrid	G	0.94	4.19	0.00	5.42	0.00	0.00	29.95
	E	50.15	84.33	88.40	58.54	24.67	45.66	35.45
	GxE	16.36	6.79	0.00	4.06	69.10	33.35	4.21
	Residual	32.55	4.69	11.60	31.99	6.23	20.98	30.39
Line	G	45.51	3.09	0.00	45.90	0.00	61.89	26.77
	E	43.15	87.59	85.78	7.66	47.68	21.88	1.96
	GxE	6.94	8.85	13.04	41.77	49.50	10.16	7.54
	Residual	4.40	0.47	1.18	4.66	2.82	6.06	63.74
OPV	G	35.09	2.49	8.96	12.06	7.69	22.74	0.00
	E	42.35	92.80	68.83	34.17	47.57	37.45	0.00
	GxE	18.13	3.57	10.91	20.07	23.17	13.67	22.64
	Residual	4.44	1.15	11.30	33.70	21.57	26.13	77.36

VC, variance component; FI, flotation index; W1000, weight of a thousand grains; TW, test weight; Ch, carbohydrates; G, genotypic effect; E, environmental effect; GxE, genotype–environment interaction effect.

**Table 4:** Average temperatures and cumulative rainfall during the critical period.

Year	RCc (mm)	RCcp (mm)	T° Minimumcp	T° Meancp	T° Maximumcp
2018	392	64.25 ± 12.14 a	15.36 ± 0.65 b	22.5 ± 0.58 c	30.26 ± 0.51 c
2019	557	109.98 ± 14.33 b	16.49 ± 0.24 d	22.11 ± 0.53 b	28.62 ± 0.71 b
2020	462	71.36 ± 42.54 a	16.07 ± 0.18 c	22.84 ± 0.23 d	30.29 ± 0.44 c
2021	588	96.16 ± 25.08 b	14.85 ± 1.03 a	20.58 ± 1.1 a	27.26 ± 1.23 a

Average values (±standard deviation) with different letters on each column for RCcp, T° Minimumcp, T° Meancp, and T° Maximumcp indicate statistical differences (Tukey’s HSD test, *p* ≤ 0.05). RCc, cumulative rainfall during the crop cycle; RCcp, cumulative rainfall in the critical period; cp, critical period.

## Data Availability

The data presented in this study are available on request from the corresponding author.
